# The Students' Guide to Dental Anatomy and Surgery

**Published:** 1877-02

**Authors:** 


*The Student's Guide to Dental Anatomy and Surgery.*—By Henry Sewill, M. R. C. S., L. D. S., etc.

The avowed object of the author of this work, which is in the form of a manual of some two hundred and three pages, is to facilitate study to the dental student, by substituting, for the voluminious literature of this branch of the science, rendered necessaary by the great progress of dentistry, a treatise in a condensed form which will prevent embarrassment, and be readily comprehended. This work is embellished with some seventy-seven illustrations handsomely bound, an l is printed on good paper with clear type.

It contains articles on the Anatomy and Histology of the Teeth, development of the Teeth, Growth of the Jaws, Dentition, Malformations and Irregularities, Caries of the Teeth, Diseases of the Pulp, Alveolar Periostitis and Abscess, Necrosis, Exostosis, Absorption of Roots, Diseases of the Gums and Mucous membrane, Abrasion and Mechanical Injuries, Salivary Calculus, Morbid Growths, Diseases of Antrum, Pivoting Teeth, Neuralgia and Diseases of the Nervous System, and finishes with Extraction of Teeth, rendering it a useful and interesting addition to the text books of the profession. Publishers: Lindsay and Blakiston, Philadelphia.

